# Designing for community engagement: user-friendly refugee wellness center planning process and concept, a health design case study

**DOI:** 10.1186/s12913-023-10007-7

**Published:** 2023-11-09

**Authors:** Ellen Solomon, Brandon Joa, Shandon Coffman, Billie Faircloth, Marc Altshuler, Bon Ku

**Affiliations:** 1 Health Design Lab, Thomas Jefferson University, Philadelphia, PA USA; 2 KieranTimberlake, Philadelphia, PA USA; 3 Department of Family and Community Medicine, Thomas Jefferson University, Philadelphia, PA USA

**Keywords:** Human-centered design, Social determinants of health, Refugees, Family medicine

## Abstract

**Background:**

Refugee and immigrant populations have diverse cultural factors that affect their access to health care and must be considered when building a new clinical space. Health design thinking can help a clinical team evaluate and consolidate these factors while maintaining close contact with architects, patients’ community leaders, and hospital or institutional leadership. A diverse group of clinicians, medical students, community leaders and architects planned a clinic devoted to refugee and immigrant health, a first-of-its-kind for South Philadelphia.

**Methods:**

The planning process and concept design of this wellness center is presented as a design case study to demonstrate how principles and methods of human-centered design were used to create a community clinic. Design thinking begins with empathizing with the end users’ experiences before moving to ideation and prototyping of a solution. These steps were accomplished through focus groups, a design workshop, and iterations of the center’s plan.

**Results:**

Focus groups were thematically analyzed and generated two themes of access and resources and seven subthemes that informed the design workshop. A final floor plan of the wellness center was selected, incorporating priorities of all stakeholders and addressing issues of disease prevention, social determinants of health, and lifestyle-related illness that were relevant to the patient population.

**Conclusions:**

Design thinking methods are useful for health care organizations that must adapt to the needs of diverse stakeholders and especially populations that are underserved or displaced. While much has been written on the theory and stages of design thinking, this study is novel in describing this methodology from the beginning to the end of the process of planning a clinical space with input from the patient population. This study thus serves as a proof of concept of the application of design thinking in planning clinical spaces.

**Supplementary Information:**

The online version contains supplementary material available at 10.1186/s12913-023-10007-7.

## Background: the design challenge

Patients from lower socioeconomic backgrounds are well known to suffer from an increased burden of acute and chronic diseases compared to the general population [1], and the adjustments that accompany refugee status or immigration can further compound health challenges [2, 3]. Even when practitioners and community leaders are united in seeking improved community health, it can be difficult to coordinate priorities to ensure a clinic addresses factors such as language differences, cultural history, nutrition, insurance, and immigration status, etc. to practice cohesive and holistic care [4]. Furthermore, the COVID-19 pandemic exacerbated health inequalities in the refugee population due to disproportionate numbers of refugees employed in designated essential industries and manufacturing, crowded housing, and lack of access to health information due to cultural and language barriers [2, 5, 6].

The City of Philadelphia is a striking demonstration of the effects of these social determinants of health. For example, people living in Philadelphia’s lower income, less healthy zip codes can see a twenty-year drop in life expectancy compared to those residing in the city’s wealthier zip codes [7]. Surveys of the city’s immigrants show most do not know which support systems to access when sick, and many overutilize emergency rooms while seldom receiving primary care [8]. Furthermore, refugee communities are a growing patient population that faces barriers to accessing health care due to cultural and language differences [9], manifesting in issues of trust in health care practitioners, logistical challenges in scheduling and transport, and health education [2].

We used design thinking methods to inform the planning goals of the first clinic in South Philadelphia dedicated to refugee and immigrant health, which opened in 2021. The concept design for the Hansjörg Wyss Wellness Center was a collaboration between the Department of Family and Community Medicine at Thomas Jefferson University Hospital, the community organizing group SEAMAAC (Southeast Asian Mutual Assistance Association Coalition), and KieranTimberlake, a Philadelphia-based architecture firm. The intended patient population was predominantly Southeast Asian, and the area around the center included people from Cambodian, Bhutanese, Burmese, Laotian, and Vietnamese backgrounds as well as Chinese, Latino, and Congolese residents. To integrate the diverse patient populations’ needs and the varying perspectives of the collaborative partners in creating a clinical space, we embraced a design thinking methodology that would best harmonize the different priorities of the organizations involved while respecting the patients’ cultural needs.

**Fig. 1:**
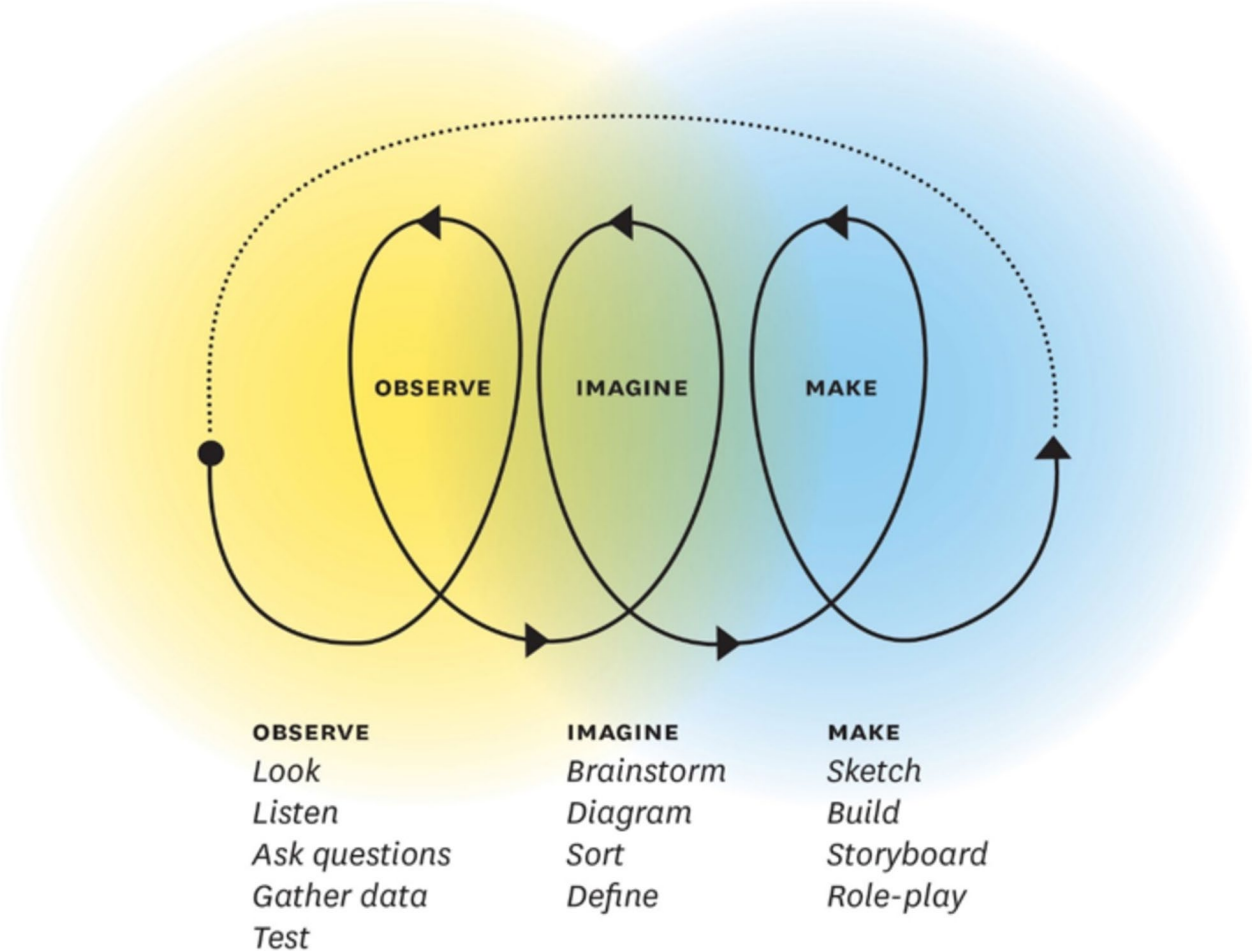
Human-centered design process. From Ku and Lupton 2020 and 2022 [10]

Design thinking is an approach to creating products and services that centers on the user experience as a source of insight [10]. A design thinking approach involves methods such as empathizing with users, rapid prototyping, and multiple rounds of testing (see Fig. 1). While many articles in the past decade have offered guidelines and theoretical explanations for applying design thinking to health care, there are few documenting how design thinking in health is concretely applied throughout the length of a specific project or intervention, especially when designing new clinical spaces or involving medical students in design solutions (see [11]).

Whereas the public is usually involved at the end of the design process for a clinical space if at all, transparency and input for the patient population were important goals from the beginning (see [12]). Clinical spaces perceived to be considerate of end user preferences can lead to greater patient satisfaction, feeling more welcome, and reduced stress for both patients and staff [13, 14]. Designing this clinic afforded a unique opportunity to observe how design thinking can integrate designer, practitioner, and patient feedback at multiple stages throughout the design process.

## Methods: the design process

### Stakeholder engagement focus groups

The design process began with developing empathy for the end-user by conducting focus groups with community members and leaders. Participants were recruited by the community organizing group through community bulletins, with the aim of reaching a qualitative understanding of participants’ social supports and stressors [1, 15]. Focus groups questions were open-ended to gather diverse insights into perceived health care access barriers and to probe desires for the new wellness center (see Supplementary material for example questions). Ten focus groups were conducted (*n* = 100 respondents) in fall 2019. Eight of these included community members of specific ethnic and linguistic groups, i.e. Mandarin Chinese, Bhutanese, Laotian, Burmese, Vietnamese, Latino, Congolese, and Cambodian; one focus group included health care and social work staff; and one focus group consisted of representatives of community organizers in the region.

Four medical students, in association with the Jefferson Health Design Lab and the Department of Family and Community Medicine, independently coded the focus group data using thematic analysis, synthesizing results by major general themes as well as specific themes per ethnic group [13, 16]. The medical students then met together to compare thematic codes and reach consensus on thematic interpretations. The results were discussed and refined in consultation with community organizers to maintain the perspectives of the members of the various cultural groups that comprised the patient populations. Qualitative data elucidated the end users’ priorities and insights, which guided the team during the design sprint and concept design phases.

### Design sprint

Using observations from the focus groups, we conducted a design workshop (“design sprint”) in September of 2019 with a diverse group of project stakeholders (architects, community workers, and clinicians) to gather insights into how cultural and socioeconomic factors could inform a design framework for a multipurpose exam room within a community wellness center. The exam room was chosen as the topic for the workshop because it represents the center of the patient experience and is an essential space for communicating to patients and family members.

First, participants were formed into groups and engaged in brainstorming sessions on designing the ideal community clinic. They were asked to write down observations of the current exam room experience (informed by personal experience and focus group data), which were recorded on sticky notes and organized into themes on white boards. These initial ideas formed the backbone for subsequent rounds of the design sprint.

After brainstorming challenges and barriers patients face with the conventional exam room design, groups were challenged to generate “How Might We” (HMW) statements. This technique of questioning helps designers to frame problems from a fresh perspective in a way that is specific and actionable [10]. Participants used the statements to distill common issues that arose in the brainstorming session.

HMW statements were shared and formed the basic scenarios for storyboards, which participants drafted to understand how the clinic might fit into their own lives and the lives of their communities. Each group chose one HMW statement to develop into “wild ideas”, i.e. without constraints of time, money, or technology for solutions, that were the topics of the storyboards. Storyboards use narratives to convey the problem identified and the possibilities for design solutions [10].

Using insights from storyboards, the groups began iterating solutions through the use of journey maps, with the realistic constraint of being implemented by the end of 2020. Journey maps are annotated timelines of the user’s experience of a proposed product. The journey map envisions the end user’s actions and experience with the product along various stages of an encounter until the product has served its use, paying attention to the actions, emotions, and physical touchpoints [10].

Virtual Reality (VR) allowed us to experience a higher fidelity iteration of the future wellness center [17]. A VR simulation presented versions of floorplans that could be seen from a bird’s-eye view and explored through a first-person perspective. The VR simulation thus demonstrated the desirability of the design features within a simulated space of the existing building. Five floor plans were drafted, featuring varying levels of alteration to the existing structure and including the earlier proposed design features to greater and lesser degrees, and a final plan was selected based on the client decision making group’s feedback from the VR simulation and floor plan diagrams.

## Results

### Focus group results

Focus groups yielded 2 major themes of “access” and “resources” and 7 subthemes shared among multiple groups, as well as priorities particular to each ethnic group (Table 1). Subthemes included language concerns, hospitality, location, flexibility, health education, cultural education, and services.

**Table 1:** Focus group themes

**Theme**	**Access**
**Subthemes**	Language barriers and need for good interpretation (8 FGs). • Lack of understanding of medical terminology (2 FGs). • Difficulty making appointments (2 FGs). • Language barriers impede health education (3 FGs). • Language differences contribute to feelings of distrust and fear of suffering from discrimination (4 FGs). Hospitality. Kind and welcoming staff are important (5 FGs). • Yelling for patients and loud noises may make some patients feel unwelcome. Location. Convenient location of the building is an asset (5 FGs). • Elders can use the space for formal and informal purposes. • First refugee wellness center in the area of South Philadelphia. Flexibility. The space should accommodate many different needs (5 FGs). • Different family arrangements will be present as well as a variety of activities.
**Theme**	**Resources**
**Subthemes**	Health education (4 FGs). • Secondary and tertiary prevention for common diseases, especially lifestyle diseases (4 FGs). • Traditional and alternative medicine is used alongside Western evidence-based medicine. • Mental health education is important (3 FGs). Cultural education. The space may facilitate intergenerational bonding and teaching (8 FGs). • Murals and public artworks can convey cultural values. Services. The space should accommodate children and have activities for the whole family (5 FGs). • Children should be able to learn about their cultural heritage. • Other issues related to social determinants of health, such as immigration status, food security, etc., can be addressed.

*FG* Focus group

### Access

The major theme of access primarily concerned barriers that patients faced to accessing clinics and organizations promoting health. The subthemes included under access were language concerns, hospitality, and location. Common to 8 groups was an emphasis on *language barriers* as a barrier to healthcare, leading to requests for in-person interpreters. Patients in these communities often rely on children or grandchildren to translate; in addition to complicating the important roles relating elders to youths in these communities, translation difficulties can lead to difficulty in making appointments, understanding medical terminology, and receiving health education, as the Laotian, Burmese, Bhutanese, and Vietnamese groups especially noted. Language barriers contributed and were related to cultural differences in communication, especially for how hospitality and honor are expressed.

Five of the groups expressed that their standards for how a *welcoming and kind staff* should treat them are important and differ from their typical prior experiences in healthcare settings. These groups noted that their ideal setting has moderate activity, space for casual conversation, and warm greetings, but typical waiting room behavior such as yelling for patients is unacceptable.

Five of the focus groups appreciated the wellness center building’s *convenient location*, as elders from the Asian communities have often been able to meet there for formal and informal social events. Jefferson Family Medicine chose the site in part because of its proximity to the planned patient populations. The wellness center’s potential to continue as a community meeting space therefore provides an opportunity to help patients feel welcome and was a prominent guideline for the team.

### Resources

Related to the previous issue of language barriers was 4 focus groups’ expressed need for *health education*, particularly on secondary and tertiary prevention for chronic conditions such as diabetes, hypertension, and hepatitis B [18]. While community members tended to be aware of many chronic diseases being an issue for their population, they lacked knowledge of disease progression and how they could expect to manage their conditions. The Laotian group in particular noted that community members’ use of traditional medicine might continue without informing Western practitioners, who are perceived as cold and condescending when patients do not understand or receive information about their illnesses. Proper presentation of education was especially heavy on the minds of this group as they recounted how three middle-aged men in their community had recently died from cardiovascular complications after being diagnosed with hypertension. The men had distrusted the advice and prescriptions of Western practitioners and had attempted to procure herbs for their hypertension, which subsequently progressed. This episode demonstrated to the team the importance of culturally sensitive health education for preserving trust in the patient-provider relationship. 3 groups also desired more education on mental health issues, noting generational differences in recognizing and seeking treatment for conditions such as depression and anxiety.

Eight groups emphasized their desire for a space that facilitates intergenerational family bonding and *cultural education*. These groups alerted the team to the need to consider how various details contribute to the space’s suitability as an intergenerational and educational meeting space: particular color combinations could either soothe or evoke unpleasant historical associations, while murals, group exercise activities such as tai chi, and traditional board games like Chinese chess could immediately provide a sense of familiarity as fixtures common to other favored meeting spaces such as parks. 5 groups wanted child services and places for children to learn about different cultures and their own ethnic group, among various *services*. Participants found child services fitting for a family medicine-run wellness center and expressed that such services would give more peace of mind if they needed to be examined on their own. Furthermore, they raised the idea that having some resources such as legal counsel or food security resources on premises, which could help coordinate strategies on social determinants of health.

### Integrating focus group data into design process

The seven themes informed the design sprint’s prototypes, which were then refined into iterations of the potential space.

**Fig. 2:**
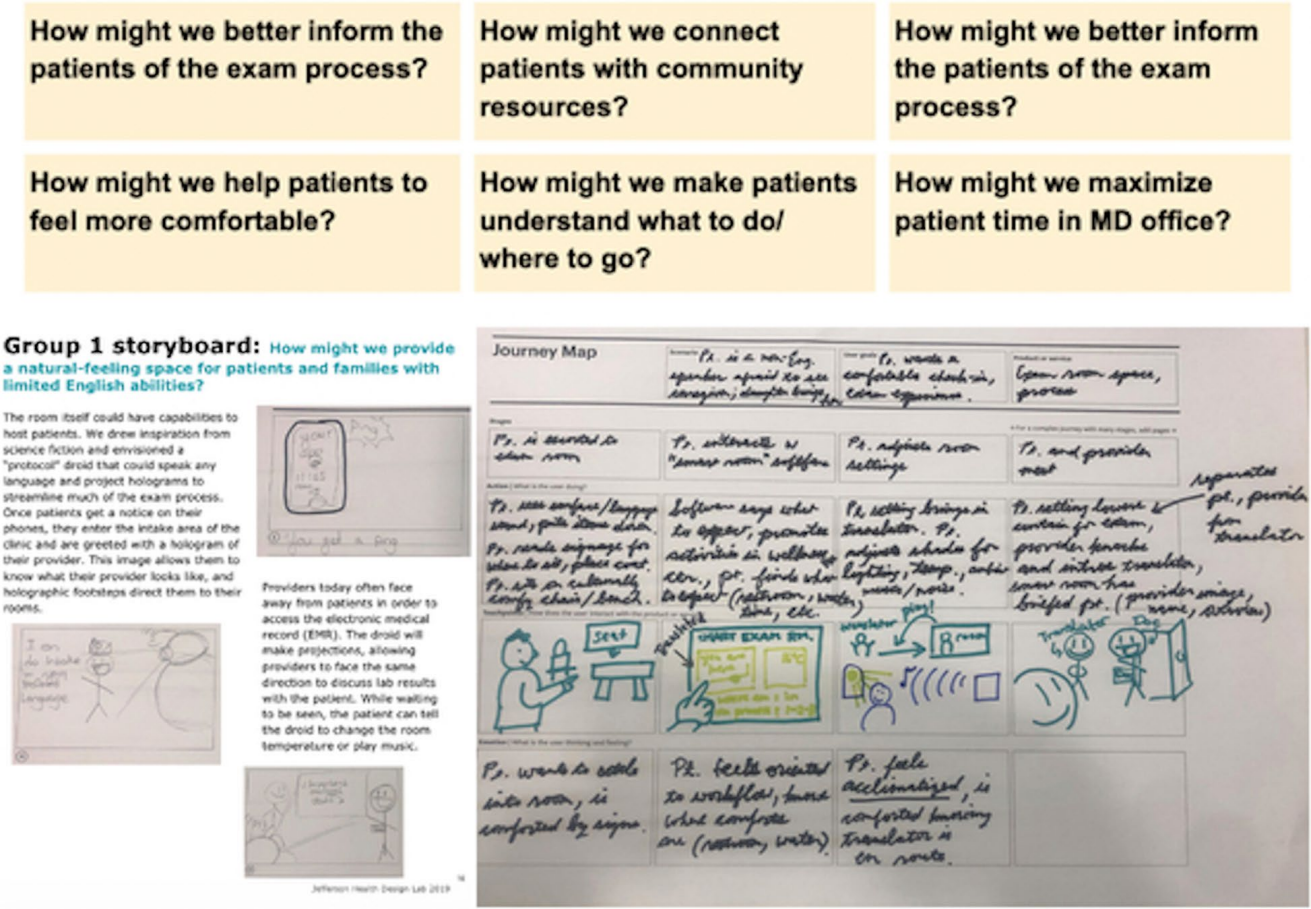
Sequence of design workshop. Brainstorming (top), HMW (“how might we”) statements (middle), storyboarding (bottom left), and journey maps

To address the need for hospitality, the team utilized the design sprint to envision spaces and processes in which rituals of continuing welcome could take place in a way similar to how spaces are used in Asian traditions (see [19]). Journey Maps conceived of the patient visit as a culturally significant ritual, mirroring patients’ own expectations towards visiting physicians (Fig. 2). For example, Chinese and Vietnamese patients had described visits to traditional medicine as experiences of hospitality; the traditional practitioner welcomes patient and family with a tea ritual and provides quiet space for relaxation, establishing trust with the patient before the visit. Taking this into account, the waiting area was configured as a library and cafe, leading into a gallery and community meeting space before the exam rooms, which were treated as intimate family meeting areas. These spaces were developed to convey a vision of professionalism less reflective of clinical distance or impassivity and more premised on showing honor to patients and respect for cultural preferences.

Recognizing the significance of the center’s location, and in order to incorporate the community members’ desire for the clinic to be a community space that promotes intergenerational bonding and facilitates health education, the design sprint envisioned space that supported modular *flexibility* to accommodate different types of activities. This included a multi-purpose space that would accommodate diverse patient needs and would allow for a sense of ownership of the space.

Acknowledging the burden of language barriers and the need to accommodate multi-generational family visits, the design sprint yielded various ideas to circumvent these barriers. “Wild ideas” including interactive an EMR that projects on the exam room wall and provides in-time translation of medical information, and Journey Maps envisioned interactive screens that provided health education videos in patients’ own languages. Rooms were envisioned as flexible spaces that allow effective placement of translators or family members who would participate in the patient encounters. Viewing the exam room as a flexible meeting space could help resolve barriers to access such as language and harsh and sterile environment.

### Final concept design

**Fig. 3:**
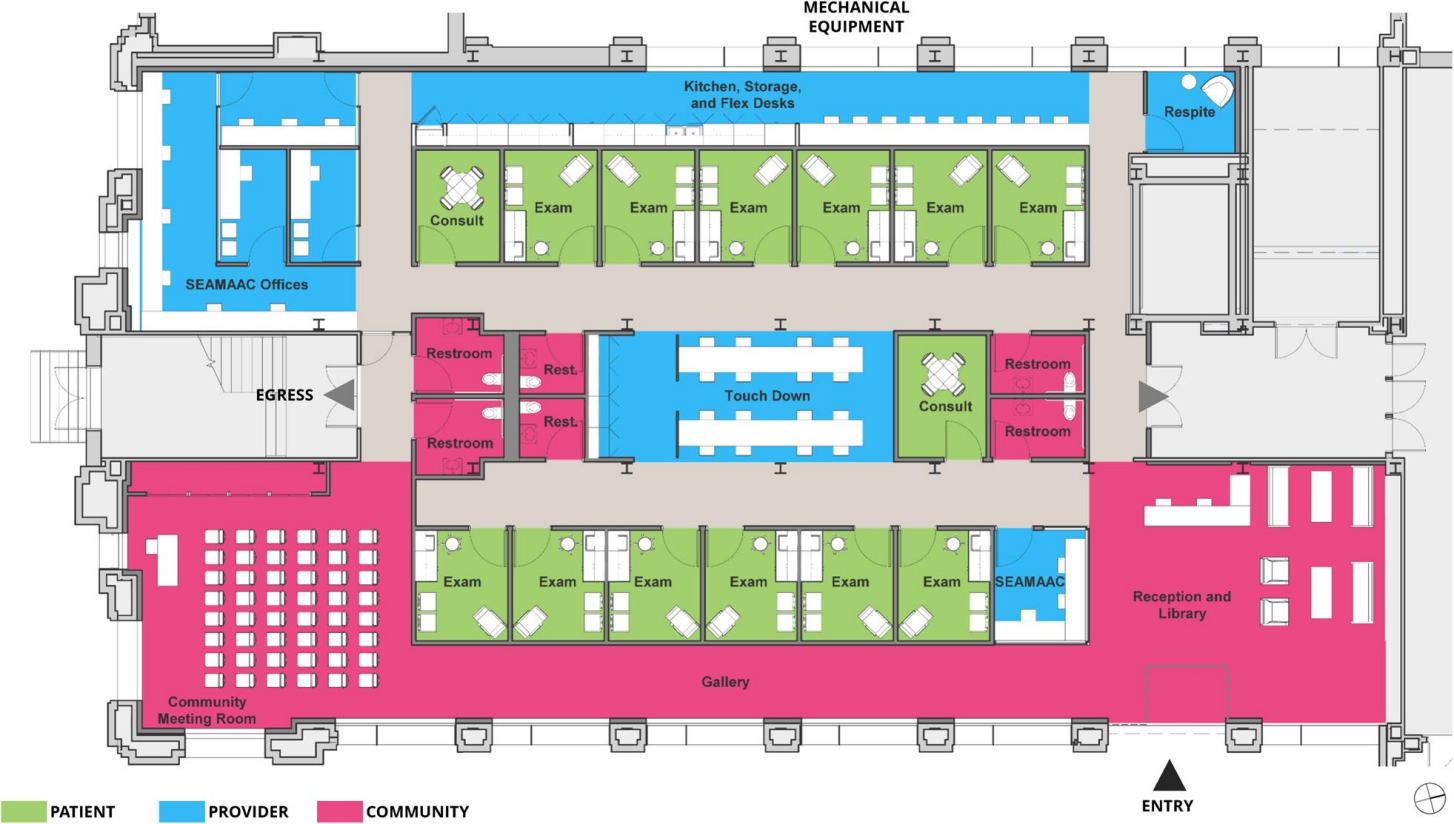
Wellness center final concept design. Patients are received in a cozy library reception area in place of a waiting room. They can proceed directly into the clinical area or move through a gallery lined with local art and cultural artifacts. The gallery opens to a multipurpose space with movable walls for community education and elder meetings, and this space leads to clinical areas and SEAMAAC offices. These adjacent areas emphasize the interconnection of physical health with sociocultural stability. Common spaces maximize natural light from tall windows with a garden view. In the center of the clinical area, staff have easy movement between patients, colleagues, and the lab. Rooms have angled computers for more face-to-face interaction, and some have movable tables. Smaller multipurpose rooms allow consultation with families, reflecting patient preferences for multigenerational family visits

Informed by the results of the focus groups and design sprint as described above, various floorplans were conceptualized, diagramed and visualized through VR technology to garner feedback from the client decision making group. A virtual first-person walkthrough let designers envision the future patients’ clinic visits and how they would interact with built-in design features like the waiting room-as-library, the gallery hallway, a multi-purpose community education space, family meeting areas, and exam rooms. The final concept design of the center was selected for its preservation of the building’s central columnar area, expected patient flow, and incorporation of innovative multipurpose spaces (Fig. 3). Spaces were color-coded and categorized according to the primary end user for which the space was designed, with green assigned to spaces for patients, blue for staff, and red for the overall community. The plan incorporated concepts from the design sprint, and represented a synthesis of the goals of hospitality, patient orientation to space, and unconventional meeting areas with the distinctive historical details that the building, a former trade school, afforded.

## Discussion

For the purposes of health services research, this study focuses on the design process itself and the ways the process elucidated and interacted with multiple stakeholders’ priorities. However, a brief discussion will help demonstrate how the final concept reflected those priorities before returning to comment on the process.

All of the focus group themes were able to be incorporated into the wellness center’s design in some form, which is evident from a walkthrough of the concept from a patient’s perspective. Upon entry, a reception and library area present the wellness center as a more welcoming place than the typical clinic. A library setting meets the need for health and cultural education, and the books and windows provide positive distractions that reduce boredom and anxiety [20]. The reception desk is placed sufficiently close to seating to show sufficient attention and preserve quiet during staff and patient interactions. Patients may enter a gallery that exhibits local art and cultural artifacts while letting in natural light from the building’s high windows. Both the gallery and the adjoining community meeting room have a view of the small community garden (not shown in the image) that can be used for health education on nutrition and healthy lifestyles. The meeting room is a flexible space that can be divided several ways and could be used for purposes such as cultural education, vaccination events, group therapy, guided exercise, etc.

Moving from the community to the patient and staff spaces, the exam rooms were planned with enough room for a few family members. With understanding that it would be difficult to have space for multigenerational families, an exam table, and ideal positioning of computers and equipment all together, consult rooms were added to allow family meetings in a less cramped setting while keeping the exam rooms from being too large. In staff areas, spaces for a community organization could make it convenient for patients to address social determinants of health during a visit. Having staff from the community organization present and proximal to the reception area was intended to promote patients’ engagement and comfortability in the wellness center overall.

### Creative solutions to challenges

Once focus groups had been conducted, one major challenge was addressing the myriad of community members’ non-clinical requests while prioritizing the primary goal of the center to meet medical needs. Some of these requests involved competing needs, such as a desire for spaces of peace and tranquility but also areas for group exercise and celebrations. Therefore, during the design sprint, several of the groups discussed the option of modular rooms that could be adapted to a variety of purposes just by rearranging some of the furniture, dividers, or accessories. Instead of attempting to fit spaces into the building for mutually exclusive purposes, allowing community members to decide when they want the spaces to fit particular needs eliminated the potential conflict between various priorities. Teams hoping to use design thinking in building clinical spaces must consider how to make the space itself adaptable to the different populations they serve that have disparate interests.

Constraints of the physical building and space were limiters on the details that could be included. Because of the building’s historical and architectural value, we agreed that many of the wall details, columns, and window configuration should be preserved, and rooms could not be as large as initially desired. Here, designing in a multidisciplinary team proved valuable, as the medical students served as communicators to nonclinical staff, architects, and community members of the clinical requirements of the exam rooms. One such point was the use of electronic medical records (EMR) during the exam coupled with placement of the computer facing away from the patient, which inhibited face-to-face rapport between the practitioner and patient. As touched upon, exam rooms were therefore designed with space for adequate computer placement, with the tradeoff of some of the rooms being unsuitable for large families to occupy all at once. To mitigate this, designated family meeting rooms were included, i.e. the consult rooms, and there were slightly fewer exam rooms in total. We perceived benefit to designing each room to solve one or two problems rather than every problem at once.

### Strengths, limitations, and future directions

The main strength of our research is documentation of each step in the design process, describing how the theory and principles of design thinking are applied in concrete settings for the benefit of multiethnic populations (including both immigrant and refugee populations) who are medically underserved and resulting in a design that harmonizes the interests of many stakeholders. The use of design thinking principles and methods with trainees to create a clinical space is novel. The methodology of arranging design thinking tools in each stage from the empathy phase (focus group analysis) to imagine (design sprint with brainstorming HMW statements) to making (journey maps, VR, and final concept) provided a high level of transparency and inclusion for the patient population communities. This process is replicable for any teams needing to harmonize the preferences of all stakeholders. Teams hoping to center the design of clinical space around end user preferences, especially for refugee, immigrant, or low-income populations, could consider holding similar design workshops with community input. Medical students in our case acted as design workshop leaders. Construction of a clinical space can present a rare and valuable opportunity for students to practice design thinking, and academic medical institutions should benefit from their contributions whenever possible. Helpful resources in design thinking included *The Field Guide to Human-Centered Design Thinking* [21] and *Health Design Thinking* [10], and our process may be considered a demonstration of those theories in health care for planning a new clinical setting.

Another strength of our research was the use of qualitative methods to gather the input of multiple end users and patient populations. Focus groups, conducted in the native language of each group, provided opportunity for community members to share barriers to engaging with a wellness center, and suggest ideas for features would promote engagement. The open-ended nature of the focus groups facilitated insightful discussions on the cultural norms such as those about hospitality or different perspectives on alternative and traditional medicine systems. Incorporated into design thinking, qualitative methods produce an invaluable account of end user priorities that reinforces the design fundamental of centering the end user’s experience. When addressing the unique needs of immigrant and refugee populations, qualitative methods allows members of these groups to make valuable contributions among the considerations of all the stakeholders.

One limitation of this work is that while this paper addresses the process by which community needs were incorporated into the design process, it does not address outcome measures or longitudinal data. While we gathered qualitative information from the focus groups to understand patients’ preferences, the Wyss Wellness Center has been operating for too short a period to collect meaningful data on the center’s effect on patients’ continuing health. The center is well positioned to collect and interpret these data in the first few years of operation using EMR and community surveys. Furthermore, having community organizers on site will be helpful for contextualizing these data within the overall needs of the communities. Specific data of interest would include incidence of cardiovascular disease and diabetes in clinic patients versus the general population in the area, typical diet habits before and after nutrition interventions, and patient satisfaction surveys. The center could also use validated measures of patients’ perceptions of quality of the space (e.g. [22]), assessing whether ongoing perceptions correspond to the completed design process. The full implications of using design thinking for planning the wellness center could require another article touching on metrics rather than the design process itself.

Another limitation of this work is that the design process described exclusively addressed the issues of community engagement from a spatial and structural point-of-view and does not address potential barriers and facilitators of community engagement from the perspective of clinical staff and health professionals. Further work should address the barriers and facilitators posed by clinical care to incorporating the community needs described here, including but not limited to use of technology to assist with communication in both reception and clinical spaces and consideration of incorporating traditional medicine practices.

Finally, a mixed strength and limitation was collection of qualitative data in a non-clinical setting, which was appropriate for a center serving the general population. We thus benefited from partnerships with other stakeholders and could consider non-clinical priorities that might affect planning. However, we were therefore unable to collect data on some clinical characteristics such as high utilizers or prevalence of specific medical conditions.

## Conclusions

Cultural considerations are as important as clinical priorities when designing a clinical space for refugee and immigrant populations, who face cultural, language, and structural barriers to accessing healthcare. Organizations may elicit these considerations and harmonize them with the priorities of all stakeholders using design thinking. In this case, barriers of language, health education, and generational differences inspired a design thinking approach that saw the clinical space as an engaging space, incorporating features such as family meeting rooms, a gallery, multi-purpose exam and waiting rooms, spaces for community use, and on-site community organizations.

A team of diverse stakeholders is critical to the design of a clinical space that meets the needs of patients, clinicians, and community members in a quickly changing health care landscape with many data sources. Health design thinking methods are adaptable for transparently engaging and harmonizing the varied interests of these stakeholders. The methods we demonstrated can be employed in a variety of projects to improve an organization’s resilience, anticipating and understanding the needs of refugee and immigrant populations such as refugees and immigrants that have already overcome many challenges.

### Supplementary Information

**Additional file 1.**

## Data Availability

The data analyzed in this study are not publicly available as they are proprietary to local community organizations, but they are available upon reasonable request to the corresponding author given appropriate ethical and community approval.

## Abbreviations

FG: Focus group
SEAMAAC: Southeast Asian Mutual Assistance Association Coalition
HMW: “How Might We” statements
VR: Virtual reality
EMR: Electronic medical record
