# Supplementary material for: Designing for community engagement: user-friendly refugee wellness center planning process and concept, a health design case study
Source: BMC Health Serv Res. 2023 Nov 9;23:1232. doi: 10.1186/s12913-023-10007-7 (PMC10636912; doi:10.1186/s12913-023-10007-7)
Supplement: Supplementary file 1 — Additional file 1. [file 12913_2023_10007_MOESM1_ESM.docx]

**Preliminary Survey Questions**

Which language(s) do you speak at home?

On a scale of 1-10, 10 being most open, how openly do you feel that you can share your health concerns with your physician or care provider? (Pick N/A if no current provider)

On a scale of 1-10, how much do you feel that your healthcare provider cares about you? Why or why not?

How often do you see a physician?

1. 0 times a year
2. 1 time per year
3. 2 times per year
4. 3+ times per year

What is your biggest barrier to taking care of your health?

Do you know where to go for help for these issues? Where would you go for these?

Legal:

Health:

Education/ESL:

Childcare:

Food security:

Employment:

What is your favorite place to spend time outside your home and work (i.e. coffee shop, friend’s house, park, river, etc.)?

What time of day are you likely to use a new wellness center in your neighborhood? (select all that apply)

1. Weekday mornings
2. Weekday afternoon
3. Weekday evening
4. Saturday
5. Sunday

What other services, besides a doctor visit, would you like to see at a wellness center? (select all that apply)

1. Eye Care
2. Foot Care
3. Dental Care
4. Physical Therapy (help recover from physical injury)
5. Acupuncture
6. Occupational Therapy (help learning everyday tasks after injury)
7. Personal counseling/couples and family therapy
8. Other ______________

What activities would you most like to see in the new wellness center?

1. Library
2. Play area for kids
3. Space for exercise
4. Outdoor garden
5. Place to host community meetings
6. Events for kids
7. Cafe
8. Other _____________

What events/ programs would you like to have at your wellness center? ( ex. Nutrition education, sex education, birth control education)

What fun activity would you like at the wellness center (i.e. coffee shop, live music, ping pong tables, chess, knitting classes, etc)?

What is your cultural background?

**Focus Group Questions:**

1. What is your biggest barrier to wellness?
2. What makes going to the doctor comfortable/uncomfortable?
3. What services are missing in your community?
4. What could we add to the wellness center that would be fun to do while waiting for services?
5. Which aspects of your culture would you like to teach and pass on to your family and children Which aspects of cultural education would you like the community to support?
6. If you could have any type of service (e.g. library, café, learning kitchen, etc.) in the wellness center, what would it be?
7. What do you think would make your wellness center feel more like home?
8. How does your community feel about the clinic building site currently?
9. Any input on the design of the building’s outside?
10. What other questions should we be asking members of your community?
